# Multicolor flow cytometry on pericardial effusion for a prompt diagnosis and treatment of hematological malignancies with heart involvement

**DOI:** 10.3389/fcvm.2022.1000259

**Published:** 2022-11-07

**Authors:** Luigi Cappannoli, Massimo Imazio, Stefan Hohaus, Gianluigi Saponara, Domenico D’Amario, Silvia Bellesi, Elena Maiolo, Marcello Viscovo, Federica Fatone, Eleonora Alma, Francesco D’Alò, Filippo Crea, Tommaso Sanna

**Affiliations:** ^1^Department of Cardiovascular Sciences, Catholic University of the Sacred Heart (UCSC), Rome, Italy; ^2^Department of Cardiovascular Sciences, Fondazione Policlinico Universitario Agostino Gemelli IRCCS, Rome, Italy; ^3^Cardiology, Department of Cardiothoracic, University Hospital “Santa Maria della Misericordia”, ASUFC, Udine, Italy; ^4^Department of Imaging Diagnostic, Radiotherapy and Hematology, Fondazione Policlinico Universitario Agostino Gemelli IRCCS, Rome, Italy; ^5^Department of Radiological and Hematological Sciences, Università Cattolica del Sacro Cuore, Rome, Italy

**Keywords:** pericardial effusion, flow cytometry—methods, malignancies, heart failure, cardiac lymphoma, guidelines

## Abstract

**Background:**

Malignancies represent 15–50% of total causes of pericardial effusions (PE). Routine analyses recommended to be performed on pericardial fluid include general chemistry, cytology, polymerase chain reaction, and microbiological cultures. Multicolor flow cytometry (FC) is a laboratory test that already proved to be useful in the detection of lymphoproliferative and metastatic malignancies in pleural and peritoneal effusions, but current guidelines do not mention its use on PE to reach a diagnosis.

**Methods:**

Our institutional protocol foresees to routinely perform a multicolor FC analysis on pericardial fluid samples obtained by pericardiocentesis, in addition to other guidelines-recommended analyses. A sample of 15–30 ml is analyzed using a lyse and wash staining method using combination panels of antibodies, allowing to detect specific cellular subpopulations, analyzing tens to hundreds of thousands of cells in few seconds. The present manuscript aims to report our single-center experience with this diagnostic tool in patients presenting with PE requiring pericardiocentesis.

**Results:**

Routine use of multicolor FC on pericardial fluid samples in our institution allowed to reach a definite diagnosis of cardiac lymphomas in two patients presenting with otherwise unexplained severe PE. This resulted in immediate start of combined immunotherapy, with patients’ clinical improvement. At 6 months follow-up both patients are alive and presented a complete disease regression.

**Conclusion:**

Preliminary evidence from routine use of multicolor FC on PE support that this is a promising tool to reach a rapid diagnosis of hematological malignancies with heart involvement, leading to a prompt initiation of targeted therapies.

## Highlights

- Flow cytometry should be considered as a routine diagnostic test every time a pericardial fluid sample is available, in addition to classical chemistry, cytology and microbiology analysis, when there is suspicion for malignant etiology.
- In patients with moderate to severe PE, rapidly worsening clinical condition and no definite etiological diagnosis, a pericardiocentesis to analyze the pericardial fluid may be considered to guide further treatments.
- Flow cytometry on pericardial (and other serous) fluid is not only useful to obtain a rapid definite diagnosis, but also to detect specific molecular therapy targets and to predict prognosis.

## Introduction

Pericardial effusion (PE) is defined as an increase in pericardial liquid (which usually amounts for 10–50 ml) due to an increased production in case of inflammatory stimuli (exudate) or to a reduced lymphatic reabsorption in case of increased systemic venous pressure (transudate) (1). PE recognizes several etiologies including infections, primary and secondary neoplasms, autoimmune diseases, uremic nephropathy, drug hypersensitivity and metabolic diseases (2). Among these, the prevalence of malignant etiologies ranges from 15 to 50% according to registries and geographic areas (1), with extra-cardiac cancers (mostly lung and breast cancer, and lymphoma) much more common than primary ones.

ESC Guidelines recommend pericardiocentesis or surgical drainage in case of cardiac tamponade, suspected bacterial or neoplastic pericarditis, or moderate to large PE not responding to conventional therapies (1). A great number of procedures are performed yearly: the National Inpatient Sample database reported 96,377 pericardiocentesis performed in the United States from 2007 to 2015, with an average of 10,710 procedures/year (3). Other sources report 32,047 pericardiocentesis performed globally in 2018 (4). The routine analyses recommended to be performed on pericardial fluid are: general chemistry (specific gravity and proteins quantification); cytology (to detect malignant cells); polymerase chain reaction (for specific infectious agents); and microbiology (aerobic and anaerobic cultures) (1–5). Cell population analysis by FC(s) is not mentioned among these analyses and therefore it is rarely performed, despite it has already proved to be useful in detection of lymphoproliferative and metastatic malignancies in pleural and peritoneal effusions (6, 7).

We report our single center experience of routine use of FC on PE samples and how this led to a definite diagnosis and a prompt initiation of a targeted therapy in two patients presenting with cardiac tamponade due to primary cardiac lymphoma.

## Methods

Our institutional protocol foresees to keep a sample (15 to 30 ml) of pericardial fluid to be analyzed by multicolor FC every time a pericardiocentesis is performed. This analysis is performed in addition to those routinely recommended (chemical-physical, cytological, PCR and microbiological analysis).

Multicolor FC is a laboratory test that uses multiple fluorescent markers and antibodies to quantify multiple parameters of a cell populations and that allows to detect specific cellular subpopulations analyzing tens to hundreds of thousands of cells in few seconds. It is performed using a lyse and wash staining method using combination panels of antibodies. Data are acquired on a FACSCantoII cytometer and analyzed using the BDFACSDiva software (BD).

The present manuscript aims to report our single-center experience with this diagnostic tool in patients presenting with PE requiring pericardiocentesis.

## Results

### Case 1

This is the case of a previously healthy 57-year-old woman who presented on April 2nd, 2021, to the E.R. of our hospital due to repetitive syncopal episodes and marked asthenia. Her medical history was remarkable for an acute mesenteritis in January 2021 occurring 2 days after the second dose of the BNT162b2 vaccine against COVID-19. At the beginning of March 2021, she also suffered from an apparently unexplained episode of acute heart failure (AHF) complicated by cardiogenic shock and transient third-degree atrioventricular (AV) block for which she underwent the following investigations: a transthoracic echocardiography (TTE) that revealed normal volumes of the left ventricle (LV) with severe systolic dysfunction, hyperechogenicity of myocardial walls and circumferential moderate PE (max 18 mm) without signs or symptoms of cardiac tamponade; an urgent coronary angiography with left and right ventricle catheterization revealing normal coronaries with LV global systolic dysfunction and postcapillary pulmonary hypertension (PAPm 28 mmHg, PAWP 21 mmHg, PVR 1.7 WU). In the suspicion of fulminant myocarditis, an endomyocardial biopsy (EMB) was also performed and high doses of methylprednisolone were administered (loading dose of 1,000 mg iv followed by 500 mg/die for 2 days) in addition to inotropes and vasopressors in intensive care unit (ICU). After 3 days the patient completely recovered, a sinus rhythm was restored and the TTE showed normal biventricular function, but with persistent PE. The results from the EMB revealed a non-specific inflammatory infiltrate with many lymphocytes and neutrophil granulocytes. A cardiac magnetic resonance imaging (cMRI) was then performed and reported hyperintensity of the lower mid-basal LV wall in T2w sequences, with hypokinesia and patchy areas of subepicardial late gadolinium enhancement (LGE) in the same area. Widespread LGE and thickening of the left atrial (LA) wall and inter-atrial septum (IAS) were also present. A positron emission tomography/computed tomography (PET/CT) with 18F-fluorodeoxiglucose (18F-FDG) showed multiple focal areas of increased metabolic activity in proximity to the superior vena cava (SVC), to the ascending aorta and to the walls of both atria and both ventricles. Further areas of metabolic activity were detected in the liver, in the right perirenal adipose tissue and in several abdominal and pelvic lymph nodes. All these findings were interpreted as suggestive for myocarditis. The patient was discharged in good clinical status with an indication to continue steroid therapy for at least 4 weeks.

**FIGURE 1:**
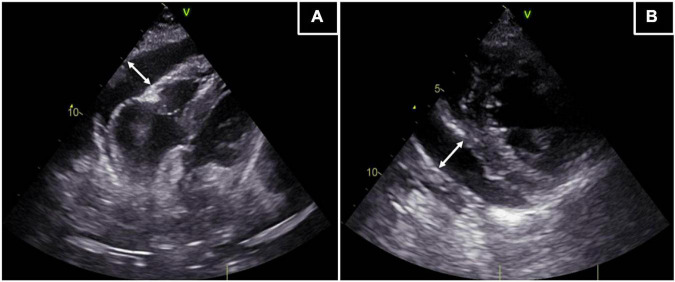
Transthoracic echocardiography. The TTE reveals a circumferential PE of moderate entity (max 17 mm). **(A)** Off-axis 4 chamber apical view of the epicardial effusion. **(B)** Parasternal short axis view of the epicardial effusion. White lines with arrows: extension of the PE.

On admission to the E.R. in April 2021 the patient presented with syncopal episodes and severe hypotension (BP 70/40 mmHg), bradycardia (HR 50 bpm), peripheral cyanosis and nausea. She was apyretic (35.5°C), serum lactates were elevated (3.5 mmol/L) and the ECG showed a complete AV block with junctional escape rhythm. The bedside TTE revealed a normal biventricular systolic function, no valvular abnormalities and persistent circumferential moderate PE (Figure 1). She was then transferred to the ICU where she was first stabilized with external pacing, volume expansion with iv crystalloids and circulatory support with dobutamine and noradrenaline infusion. The suspected diagnosis was an early relapse of acute AHF due to the myocarditis and, therefore, high doses of corticosteroids were newly administered (methylprednisolone 500 mg iv/daily), with restore to sinus rhythm but only partial improvement of clinical conditions. In the following days a cMRI was repeated and it showed an increase of the already known expansive/infiltrative tissue surrounding the SVC, the aortic root, the roof and posterior wall of both the atria and the IAS; there was also an extension of disease in the basal interventricular septum (IVS). A second EMB was then performed to obtain an etiological diagnosis trying to collect samples from the atrial walls (the ones mostly involved), but the result was once again a non-specific inflammatory aggregate of large leukocytes, with numerous granulocytes and very few myocardial cells. Furthermore, a bone marrow biopsy was performed, and it resulted negative for infiltration by clonal or malignant cells.

**FIGURE 2:**
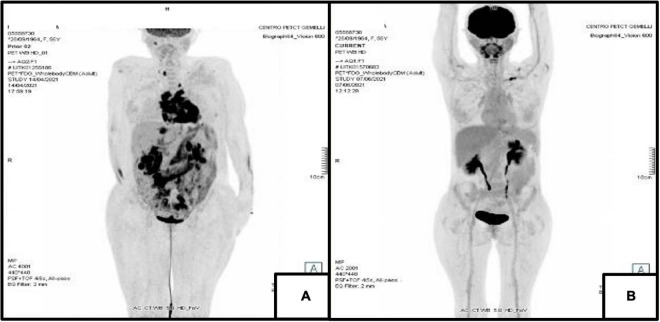
18F-FDG Positron Emission Tomography. **(A)** PET/CT of April 2021 showing disease with intense metabolic activity in the following areas: SVC, aortic root, pulmonary artery, both atria, IAS, IVS and LV in the thorax and liver, both kidneys, mesenterial adipose tissue, annexes and several lymph nodes in the abdomen. **(B)** PET/CT of June 2021 showing an almost complete resolution of pathological metabolic activity both in the thoracic and abdominal previous sites of uptake.

After another week of stay in ICU with supportive therapy and no further improvement of the clinical status, the patient suffered from another episode of complete heart block and hemodynamic instability, so that a bicameral pacemaker was urgently implanted. She underwent another PET/CT, that confirmed the already known multiple areas of intense metabolic activity surrounding the SVC, aortic root, pulmonary artery, both atria, IAS, IVS and LV, but with an increased extension compared to the previous PET/CT of March. The multiple lesions in the abdomen had also increased (Figure 2A).

The PE progressively increased despite medical therapy, leading to cardiac tamponade, so that an urgent evacuative pericardiocentesis was performed with drainage of about 650 ml of serous fluid and almost complete resolution of the effusion at TTE. The pericardial fluid was sent for chemistry and cytological analysis, without leading to an etiological diagnosis.

**FIGURE 3:**
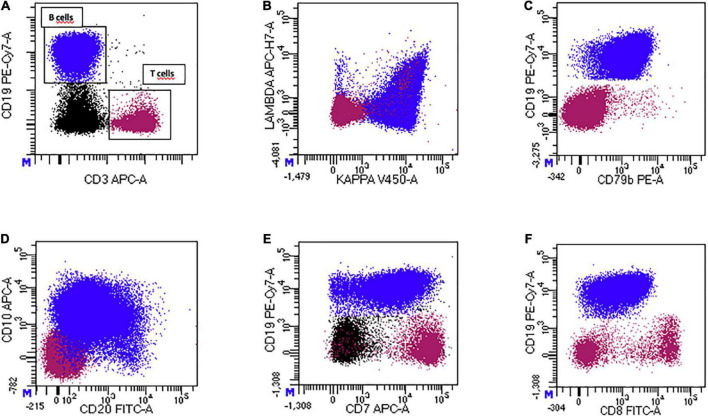
Multicolor flow cytometric analysis of pericardial fluid of pt 1. **(A)** CD19 + B-cell population and CD3 + T-cell population (internal negative control). **(B)** The CD19 + B-cell population shows a pathological clonal restriction for surface kappa light chain. **(C)** The CD19 + B-cell population is positive for CD79b, a pan-B-cell marker. **(D)** Expression of CD20 (dim-expression) and CD10 (intermediate-expression) on the pathological CD19 + B-cell population. **(E,F)** Aberrant expression of the T-cell markers CD7 and CD8 on the pathological CD19 + B-cell population.

A sample of the fluid also underwent a multicolor FC analysis (Figure 3). We detected an aberrant, clonal, mature CD19 + B cell population that constituted 58% of total events. The CD19 + cells showed a clonal restriction for kappa light chains, expressed CD10, CD79b and sIgM. CD20 was expressed with a low level of intensity, while the precursor antigen CD34 was absent. Assessing T cell markers, we observed a strong CD7 expression on the clonal B cell population, while other markers, such as CD5, sCD3, CD4 and CD8 were negative. Intracytoplasmic staining confirmed the B cell origin of the aberrant population. The aberrant population stained positive with cyCD79a and negative with CyCD3. Physical parameters of the aberrant B cell population were compared to a reactive CD3 + CD5 + CD7 + T population that constituted 25% of total events. B cells with the aberrant phenotype were of larger size and had a higher cellular complexity compared to normal T lymphocytes. These characteristics were suggestive for a diagnosis of an aggressive CD10 + large B cell lymphoma with a peculiar aberrant expression of the CD7 antigen.

The patient immediately started a combined immunochemotherapy with rituximab, fractioned cyclophosphamide, methotrexate, dexamethasone, cytarabine and etoposide (GMALL protocol).

The clinical conditions of the patient rapidly improved, and 3 weeks after start of treatment she was discharged with indication for further polychemotherapy cycles. At follow-up in June 2021, PET/CT revealed a marked reduction in metabolic activity both at cardiac level and in the abdominal areas of previous uptake (Figure 2B). A PET/CT performed in October 2021 confirmed complete metabolic remission.

### Case 2

This is the case of a 78-year-old woman with medical history unremarkable except for arterial hypertension, gallbladder stones, previous hemithyroidectomy due to a benign nodular thyreopathy and anxious-depressive syndrome. The patient suffered from a persistent dry cough since December 2021, for which she took NSAIDs with only partial relief. Due to persistence of symptoms and new onset of dyspnea on exertion (NYHA II-III) she presented herself to our E.R. on January 20th, 2022. On admission, she was asymptomatic at rest for chest pain and palpitations, mildly dyspnoic and tachypneic at rest (respiratory rate of 20 acts per minute). Blood pressure was 100/60 mmHg with a heart rate of 97 bpm. Mild bilateral ankle edema was also present. Blood tests revealed a hemoglobin of 11.8 g/dl, white blood cells 8.83 × 10^9^/L, platelets 363 × 10^9^/L, normal hepatic and renal function, lactic dehydrogenase 238 mU/ml (n.v. < 250 mU/ml), C-reactive protein 16 mg/dl (n.v. < 5 mg/dl) and Nt-proBNP 1771 pg/ml (n.v. < 125 pg/ml).

The chest x-ray depicted no signs of pulmonary edema, nor pleural effusion or cardiac enlargement. A TTE was then performed, and it documented a severe circumferential PE (max 30 mm antero-diaphragmatic) with 2D and Doppler signs of ventricular filling obstruction (right atrium and ventricle diastolic collapse, dilated with reduced collapse inferior vena cava and pulse Doppler transmitral inflow respiratory variability of 50%). A chest contrast CT scan confirmed the PE, but also revealed multiple lymphadenopathies in the mediastinum with a maximum length of 16 mm.

**FIGURE 4:**
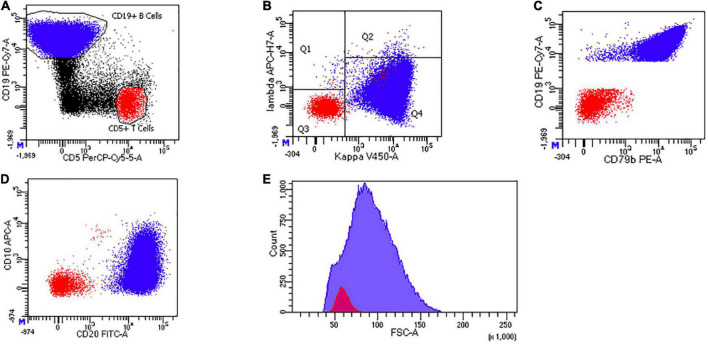
Multicolor flow cytometric analysis of pericardial fluid of pt 2. **(A)** CD19 + B-cell population and CD5 + T-cell population (internal negative control). **(B)** The CD19 + B-cell population shows a pathological clonal restriction for surface kappa light chain. **(C)** The CD19 + B-cell population is positive for CD79b, a pan-B-cell marker. **(D)** Expression of CD20 (bright-expression) and CD10 (partial and heterogeneous expression) on the pathological CD19 + B-cell population. **(E)** Cell size (FSC-A) distribution of CD19 + B cells and CD5 + T cells. CD19 + pathological population is larger than normal CD5 + T lymphocytes.

The patient was therefore admitted to the ICU and, on January 21st, she underwent an urgent pericardiocentesis with drainage of about 700 cc of hematic fluid. We sent samples for general chemistry, cytology, and microbiology as per current Guidelines. All these analyses resulted non-diagnostic. We also sent a sample of the pericardial fluid to the hematology laboratory for immunophenotyping through multicolor FC using the same methodology described in “Case 1” (Figure 4): we observed the presence of a CD19 + CD 20 + CD79b + mature B lymphocyte population, of medium-large size and increased cellular complexity, partially positive for CD10, with clonality for Kappa light chains. This population represented the 82% of the total cellularity of the pericardial fluid.

Also in this case, FC succeeded in providing the diagnosis of a B cell Non-Hodgkin lymphoma with a primary pericardial location, that otherwise would have gone undiagnosed. Both contrast-enhanced CT and PET/CT failed to show any morphological alterations or areas of increased metabolic activity both in the mediastinum and abdomen.

Based on the detection of the clonal B cell population in the pericardial fluid, the patient started combine immunochemotherapy with rituximab, cyclophosphamide, vincristine and prednisone (R-CVP regime). Therapy was well tolerated. She was discharged in good clinical conditions, with no residual PE, and with indication to continue therapy with further polychemotherapy cycles.

## Discussion

We present two cases of patients with large PE leading to cardiac tamponade requiring urgent pericardiocentesis. PE is a relatively common condition and, despite its diagnosis is quite simple, based on signs and symptoms at physical examination, on TTE detection of fluid between the pericardial sheets, and on cMRI and CT scan in selected cases (8), the exact etiology of pericardial disease remains often unresolved. This in fact could require an analysis of the fluid invasively obtained by a surgical drainage or a percutaneous pericardiocentesis (5) and, even in these cases, the routine examinations performed on it could give negative or non-specific results. In both presented cases the currently recommended analyses on pericardial fluid were indeed unhelpful to obtain a definite etiological diagnosis, which could be reached only after performing multicolor FC.

Multicolor FC is an innovative but consolidated technique that uses multiple fluorescent markers and antibodies to detect specific cellular subpopulations in samples usually obtained from percutaneous aspiration (liquid) or core needle biopsy (solid), allowing a rapid analysis of tens to hundreds of thousands of cells. It detects the presence of a selected cellular population (e.g., neoplastic) in the sample and also can study the expression of different antigens in those cells (9). In our patients multicolor FC of pericardial fluid detected the presence of a clonal B cell population suggestive for lymphoma: in the first case an aggressive CD10 + large B cell lymphoma with a peculiar aberrant expression of the CD7 antigen, and in the second case a medium to large-sized B cell lymphoma with partial expression of CD10, and a primary pericardial location. It must be pointed out, that histological examination of a tissue biopsy is the gold standard for diagnosis and classification of lymphoproliferative diseases. However, our description of the two cases illustrates clinical situations in which FC helped to establish a diagnosis of lymphoproliferative disease. In the first case, histological examination of bioptic samples of the myocardium did not yield a diagnosis, and the severe clinical conditions did not allow for further and more invasive diagnostic procedures to obtain additional tissue material. In the second case, the lymphoproliferative disease was a primary effusion lymphoma limited to the pericardial fluid. In both cases FC allowed a rapid diagnosis in a few hours with the possibility to immediately start therapy as clinical conditions of the patients were severe. Moreover, even the cytology performed on the pericardial fluid failed to detect the malignant cell population in both patients’ samples, despite a high sensitivity (90%) is reported for this kind of analysis (10). On the contrary, the identification of cancer cells by multicolor FC confirmed the evidence that this latter is a more rapid, reproducible, and even sensitive method for detecting specific cellular populations (11).

FC is not only useful to obtain a definite diagnosis, but also might be helpful in rapidly identifying aberrant antigen expression or expression of target antigens for therapy. Aberrant expression of T cell markers can be occasionally found in diffuse large B cell lymphomas (DLBCL) (12). Among these, CD5 is the most frequently expressed T cell antigen, found in about 15% of DLBCL cases, while other aberrant T cell markers including CD7 are expressed in less than 5% of cases. Aberrant expression of T cell markers in B cells lymphomas has been associated with a worse prognosis (12). The expression of CD7 by the lymphoma of the first patient was indeed suggestive for an aggressive form, needing a rapid and more intensive treatment.

The use of multicolor FC has an already well-established role in in the diagnosis of lymphoproliferative disorders from pleural and peritoneal fluids (6–7), while its use on pericardial fluid has been much less described, mainly in single case reports (13, 14). Our cases contribute to these few data, and we confirm that FC is a very useful technique to guide clinical choices. In particular in the first case, FC eventually helped to establish a diagnosis of aggressive B cell lymphoma after several other investigations (some of which invasive) failed and clinical conditions of the patients continued to worsen. The rapid identification of the aberrant B cell population allowed the prompt start of immunochemotherapy in these critically ill patients and the treatment led to a rapid clinical improvement.

In this paper we reported our institutional experience with a systematic use of multicolor FC performed on PE, but with recognize that this approach would probably be not feasible in every hospital due to cost-effectiveness issues. When FC can’t be performed on each PE as routine test, it may have an important role at least for those patients with a high suspicion of malignant etiologies (for example when the drained fluid is frankly hematic, or the patient has other signs of cancer disease) and in those without a pretest probable alternative diagnosis.

In conclusion, FC on pericardial fluid obtained by pericardiocentesis or surgical drainage can lead to a prompt and definite diagnosis of lymphoproliferative disorders (and other malignancies), especially in those cases without involvement of any other body sites and those cases with failed bioptic approaches or difficult sites of access for biopsy. As a limitation of our study, we recognize that a sample size of two patients is too small to assess a definite conclusion, but our experience, summed to those previously reported in literature on pericardial and other serous fluids, supports the possible implementation in future guidelines of FC among the routine analyses to be performed on pericardial fluid (Supplementary Table 1). This could significantly reduce the “time-to-diagnosis” and, therefore, the “time-to-treatment” for patients with suspected malignant etiology and urgent need for therapy.

## Data availability statement

The raw data supporting the conclusions of this article will be made available by the authors, without undue reservation.

## Ethics statement

Ethics review and approval was not required for the study on human participants in accordance with the local legislation and institutional requirements. The patients/participants provided their written informed consent to participate in this study. Written informed consent was obtained from the individual(s) for the publication of any potentially identifiable images or data included in this article.

## Author contributions

LC, TS, and GS conducted the study. SB, EM, MV, and FF supported the realization of the study. LC and MI had a leading role in writing the manuscript. SH and DD’A had a leading role in manuscript revision. EA, FD’A, FC, and TS had a supporting role in manuscript revision. All authors have read and agreed to the content of the manuscript.
