# Supplementary material for: Multicolor flow cytometry on pericardial effusion for a prompt diagnosis and treatment of hematological malignancies with heart involvement
Source: Front Cardiovasc Med. 2022 Nov 7;9:1000259. doi: 10.3389/fcvm.2022.1000259 (PMC9676641; doi:10.3389/fcvm.2022.1000259)
Supplement: Supplementary file 1 [file Table_1.docx]

**SUPPLEMENTS**

**Supplementary Table 1. Proposed routine analysis to be performed on pericardial fluid.**

(This table has been modified from Adler Y, Charron P, Imazio M, Badano L, Barón-Esquivias G, Bogaert J. 2015 ESC Guidelines for the diagnosis and management of pericardial diseases. 2015. Eur Heart J. Nov 7(42): 2921-2964. doi:10.1093/eurheartj/ehv318)

| **ANALYSIS** | **TEST** | **ETIOLOGY** |
| --- | --- | --- |
| General chemistry | Specific gravity > 1.015  Protein level > 3 g/dL  Protein fluid/serum ratio > 0.5  LDH > 200 mg/dL  Fluid/serum ratio > 0.6  Glucose  Blood cell count | Exudate |
| Cytology | Cytology (higher volumes of fluid, centrifugation, and rapid analysis improve diagnostic yield) | Cancer |
| Polymerase chain reaction (PCR) | PCR for specific infectious agents (i.e. TBC) | Infective |
| Microbiology | Mycobacterium, aerobic and  anaerobic cultures. | Infective |
| Multicolor Flow Cytometry (FC) | FC using different antibodies to detect clonal cell population and specific CD. | Cancer |

CD = cluster of differentiation; TBC = tuberculosis.
